# Monitoring the health of transgender and other gender minority populations: Validity of natal sex and gender identity survey items in a U.S. national cohort of young adults

**DOI:** 10.1186/1471-2458-14-1224

**Published:** 2014-11-26

**Authors:** Sari L Reisner, Kerith J Conron, Laura Anatale Tardiff, Stephanie Jarvi, Allegra R Gordon, S Bryn Austin

**Affiliations:** Department of Epidemiology, Harvard School of Public Health, Boston, MA USA; The Fenway Institute, Fenway Health, Boston, MA USA; Department of Health Sciences, Northeastern University, Boston, MA USA; Institute for Child, Youth, and Family Policy, Heller School for Social Policy and Management, Waltham, MA USA; Channing Division of Network Medicine, Department of Medicine, Brigham and Women’s Hospital, Harvard Medical School, Boston, MA USA; Department of Psychology, Suffolk University, Boston, MA USA; Department of Social and Behavioral Sciences, Harvard School of Public Health, Boston, MA USA; Division of Adolescent and Young Adult Medicine, Boston Children’s Hospital, Boston, MA USA; Department of Pediatrics, Harvard Medical School, Boston, MA USA

**Keywords:** Transgender, Health surveys, Measurement, Methods

## Abstract

**Background:**

A barrier to monitoring the health of gender minority (transgender) populations is the lack of brief, validated tools with which to identify participants in surveillance systems.

**Methods:**

We used the Growing Up Today Study (GUTS), a prospective cohort study of U.S. young adults (mean age = 20.7 years in 2005), to assess the validity of self-report measures and implement a two-step method to measure gender minority status (step 1: assigned sex at birth, step 2: current gender identity). A mixed-methods study was conducted in 2013. Construct validity was evaluated in secondary data analysis of the 2010 wave (*n* = 7,831). Cognitive testing interviews of close-ended measures were conducted with a subsample of participants (*n* = 39).

**Results:**

Compared to cisgender (non-transgender) participants, transgender participants had higher levels of recalled childhood gender nonconformity age < 11 years and current socially assigned gender nonconformity and were more likely to have ever identified as not completely heterosexual (*p* < 0.001). No problems with item comprehension were found for cisgender or gender minority participants. Assigned sex at birth was interpreted as sex designated on a birth certificate; transgender was understood to be a difference between a person’s natal sex and gender identity. Participants were correctly classified as male, female, or transgender.

**Conclusions:**

The survey items performed well in this sample and are recommended for further evaluation in languages other than English and with diverse samples in terms of age, race/ethnicity, and socioeconomic status.

## Background

The term *gender minority* refers to transgender and gender nonconforming people whose sex assigned at birth is different from their current gender identity or expression. Gender minorities appear to be disproportionately affected by adverse health outcomes compared to cisgender (i.e., non-gender minority) people [1–7]. A key barrier to monitoring the health of gender minority populations is the lack of brief, validated tools with which to identify participants in health research [8, 9]. The current study aims to fill this methodological gap.

A two-step method for identifying gender minority participants has been proposed [10–13]. This procedure uses natal sex (step 1) and current gender identity (step 2) to cross-tabulate natal sex/gender status. We use the term *natal sex/gender status* to refer to the biological and social cross-classification of participants based on assigned sex at birth (natal sex) and current gender identity (gender) that allows measurement of gender minority and cisgender identities. We use the word *status* because statuses can change over time and people’s gender identities can and do shift across the lifecourse. A strength of the two-step approach is that it takes into account both natal sex (biological) and gender (social) processes, which are key for epidemiologic studies of health [14–18].

However, validated natal sex and current gender identity measures are needed to implement a two-step method in population-based health research and health surveillance. In cross-sectional data systems where there is no previous information available about natal sex of participants, both birth sex and current gender identity items are necessary to classify participants as transgender or cisgender. Examples in the U.S. include the National Health Interview Survey, the Behavioral Risk Factor Surveillance System, the National Health and Nutrition Examination Survey, and the Youth Risk Behavior Surveillance System. Longitudinal epidemiologic research structures often have birth sex reported by parents or a medical provider at enrollment. Examples are longitudinal cohort studies (e.g., the Avon Longitudinal Study of Parents and Children) and cohort-related surveillance systems (e.g., U.S. Veteran’s Administration medical records system). In such data structures only a validated current gender identity item would be needed to capture natal sex/gender status and begin monitoring the health of gender minorities in these cohorts.

The goal of this research was to use a unique mixed-methods design to evaluate the validity of self-report natal sex and gender identity survey items using the Growing Up Today Study 1 (GUTS1), a longitudinal cohort study of U.S. young adults. In Phase 1, secondary data analysis was conducted with the 2010 GUTS1 wave to quantitatively evaluate measurement validity. We hypothesized that participants classified as gender minority on the basis of discordant natal sex and current gender identity would report higher levels of childhood gender nonconformity, higher levels of current socially assigned gender nonconformity, and greater likelihood of ever having identified as a sexual minority (i.e., not completely heterosexual) in their lifetime relative to cisgender participants. In Phase 2, cognitive testing interviews were conducted with a subsample of GUTS1 participants to qualitatively assess performance of natal sex and current gender survey items. To our knowledge, this is the first peer-reviewed evaluation of any natal sex and current gender identity measures in a national study of young adults.

## Methods

### Participants

GUTS1 is a national longitudinal cohort of children of participants of the Nurses’ Health Study II, a prospective cohort of female registered nurses across the United States. At enrollment in 1996, the GUTS1 sample consisted of more than 16,000 youth ages 9 to14 years (7,843 males and 9,039 females) [19]. GUTS1 participants have completed surveys assessing their health approximately every two years since 1996. The institutional review board at Brigham and Women’s Hospital approved the GUTS1 study and Phase 1 and 2 of this project.

### Survey items for validation

Sex of GUTS1 participants was reported by their mothers at baseline in 1996. The current study was designed to validate two self-report measures. First, a natal sex measure, drawn from the largest survey of U.S. transgender adults to date [5, 20]: “What sex were you assigned at birth, on your original birth certificate? (check one)” with response options “Female” and “Male”. Second, a gender identity measure added to the 2010 GUTS1 questionnaire [21]: “How do you describe yourself?” with response options “Female”, “Male”, “Transgender”, and “Do not identify as female, male, or transgender”.

### Phase 1: quantitative evaluation of validity

#### Sample

To be included in the analytic sample for Phase 1, GUTS1 participants had to complete the 2010 survey wave and selected measures from the 2005 wave (*n* = 7,831). Sociodemographics of the data analytic sample were compared to the original baseline GUTS cohort (*n* = 16,882). Respondents did not differ significantly on age in 1996 or race/ethnicity; however, a higher proportion of young people in the data analytic sample were female (maternal-reported sex) compared to the original baseline cohort.

#### Measures

Recalled childhood gender nonconformity was used for criterion-related validity (i.e., demonstrating an empirical association with some criterion or “gold standard”) [22, 23]. In 2005, four items taken from a validated questionnaire [24] assessed gendered behavior as a child (up to age 11) with respect to play, toys, and gender self-perception. All responses used a 5-point scale ranging from always boys/masculine to always girls/feminine, with “not applicable” responses coded as systematic missing. Items correlated from Pearson *r =* 0.47-0.63 (*p* < 0.0001). The items were added so that higher scores indicated greater childhood gender nonconformity according to natal sex (Cronbach’s α = 0.83). Sex-specific scores were coded categorically such that participants were classified as high (at or above top decile score; score ≥9 for natal males and ≥12 for natal females), moderate (below top decile, at or above median, below top decile; score 6–8 for natal males and 8–11 for natal females), and low (below median; score <6 for natal males and <8 for natal females) in recalled childhood gender nonconformity. These cut-points were selected to be consistent with previous research using this scale [25, 26].

Socially assigned gender nonconformity (how you think others perceive you) was assessed in 2010 with a brief two-item validated measure [27] and used to evaluate construct validity (i.e., demonstrating the measure behaves as expected relative to established measures of other relevant constructs) [22]. The items asked about “appearance, style, or dress” and “mannerisms” each on a seven-point Likert-scale ranging from very feminine to very masculine and were highly correlated (Pearson *r* = 0.68; *p* < 0.0001). Responses to the items were recoded so that a value of 1 corresponded to gender expression that was very conforming to the participants’ birth sex and a value of 7 indicated the participants’ gender expression was very non-conforming relative to their birth sex. Response scores were added so that higher scores indicated greater gender nonconformity relative to natal sex (Cronbach’s α = 0.81). Sex-specific scores were categorized as high (at or above top decile score ≥5 for natal males and ≥6 for natal females) or low (score <5 for natal males and <6 for natal females).

Sexual orientation was used to evaluate known-groups validity (i.e., demonstrating the measure can differentiate members of one group from another) [22] because clinical practice with transgender patients suggests sexual identity development and questioning is often a starting point for transgender identity emergence [28]. Participants were asked in 2010 whether they had ever in their lifetime identified as lesbian/gay, bisexual, or mostly heterosexual (i.e., sexual minority identity). Information on age, education, and race/ethnicity was also collected.

#### Data analysis

A conceptual overview of natal sex/gender status measurement in GUTS1 is presented in Table 1. Natal sex/gender status was coded as follows: (1) “Male”: male baseline sex and selected “Male” on the gender identity 2010 item; (2) “Female”: female baseline sex and selected “Female”; and (3) “Gender Minority”: selected “Transgender,” “Do not identify as male, female, or transgender,” or a cross-sex identity (e.g., male baseline sex and selected “Female” in 2010). All gender minority participants were examined in aggregate to maximize statistical power for comparisons.

**Table 1:** **Conceptual overview natal sex and current gender identity measurement using a two-step method in the growing up today study 1**

**STEP 1: SEX**		
*What sex were you assigned at birth, on your original birth certificate? (check one)*		
Female		
Male		
**STEP 2: GENDER IDENTITY**		
*How do you describe yourself? (check one)*		
Female		
Male		
Transgender		
Do not identify as female, male, or transgender		
	Assigned sex*	
	**Male**	**Female**
	(maternal-reported male sex on original birth certificate)	(maternal-reported female sex on original birth certificate)
	
**Current gender identity**		
Male	**Cisgender male** ± (male birth sex, male gender identity)	**Cross-sex male identity** (female birth sex, male gender identity)
	
Female	**Cross-sex female identity** (male birth sex, female gender identity)	**Cisgender female** (female birth sex, female gender identity)
	
Transgender	**Transgender identity** (male birth sex, transgender identity)	**Transgender identity** (female birth sex, transgender identity)
	
Do not identify as male, female, or transgender	**Do not identify** (male birth sex, some other diverse gender identity)	**Do not identify** (female birth sex, some other diverse gender identity)
	

*Infants born intersex are assigned either a female or male birth sex by a medical provider at birth.

± The term “cisgender” is used to refer to non-transgender males and females. The prefix “cis-” in Latin means “on this side of”, opposed to *trans* or *ultra*, across, beyond. *Transgender* is an umbrella term used to describe people whose sex assigned at birth is incongruent or different from their current gender identity or expression.

SAS v9.3.1 was used for all statistical analyses. The univariate distribution of all variables was examined by natal sex/gender status. Due to small cell sizes, analyses were descriptively focused. Proportional differences in high recalled childhood gender nonconformity, high current gender nonconformity, and lifetime report of sexual minority identity by natal sex/gender status were tested using Fisher’s exact tests. Analysis of variance (ANOVA) was used to compare mean differences in age by natal sex/gender status.

### Phase 2: cognitive testing with a subsample of GUTS1 participants

#### Sample

Between November 2012 and April 2013, 39 GUTS1 participants completed a cognitive testing interview via telephone (16 cisgender female, 14 cisgender male, 9 gender minority). The initial Phase 2 sample included all 2010 GUTS1 participants who: (1) indicated a gender minority status (selected “Transgender”, “Do not identify” or a cross-sex identity, or who contacted GUTS administrators between 1996–2010 to request that they receive a survey for the other gender); (2) indicated cisgender status (reported a 2010 gender identity concordant with baseline sex); (3) skipped the gender identity measure (all were cisgender; n = 4). Stratified disproportionate sampling (sampling fraction varied across groups) was used to select the sample. Invited to take part in the cognitive testing substudy were 116 GUTS1 participants; 41 responded (response rate = 35%). Interviewing was stopped when saturation was reached (n = 39) [29].

#### Procedures

We utilized a retrospective talk-aloud method [30], which has been used widely in survey item validation [21, 27, 31, 32]. Participants completed a brief self-report questionnaire including survey items on natal sex and gender identity. We then conducted individual, semi-structured qualitative interviews to probe question-response processes. Scripted and unscripted interviewer probes were used to clarify item interpretation, identify difficulties with comprehension, and assess respondent burden [33]. Interviews were conducted in English, averaged 25 minutes, and were digitally recorded. Participants received a $25 gift card for participating.

#### Data analysis

Cognitive interviews were analyzed consistent with best practices in the field [30, 33–36] and previous research [21, 27, 31, 32]. Transcripts were analyzed independently by two study team members. Analyses were structured around four thematic areas: item interpretation, item clarity, item response options, and emotional reactions relevant to study aims. Particular attention was paid to any problems that might negatively impact the accuracy and completeness of data. Common responses were grouped into categories relevant to the four thematic areas. Coding inconsistencies were resolved through discussion among the study team.

## Results

### Phase 1: quantitative analysis of validity

Table 2 presents natal sex/gender status by maternal-reported sex (1996). Table 3 presents the distribution of demographics and other characteristics used to evaluate validity by natal sex/gender status. Criterion-related validity of natal sex/gender status was supported: as predicted, gender minority participants scored higher on recalled childhood gender nonconformity compared to cisgender females and males. Construct validity was supported in that gender minority participants had higher levels of current socially assigned gender nonconformity than cisgender female and male participants. Known-groups validity was supported in that a significantly higher proportion of gender minority participants, compared with cisgender females and males, reported having ever identified as a sexual minority in their lifetime.

**Table 2:** **Phase 1: natal sex and current gender identity cross-classified using a two-step method among U.S. young adults in the growing up today study 1**

	Maternal-reported natal sex (1996)		
	Female (n = 5226)	Male (n = 2605)	Total sample (n = 7831)
	*% (n)*		
Current gender identity			
Cisgender female	99.69 (5210)	0.00 (0)	66.53 (5210)
Cisgender male	0.00 (0)	99.62 (2595)	33.14 (2595)
Gender minority (Cross-sex identified, transgender, or do not identify)	0.31 (16)	0.38 (10)	0.33 (26)
Cross-sex identified	0.06 (3)	0.15 (4)	0.09 (7)
Transgender	0.06 (3)	0.08 (2)	0.06 (5)
Do not identify as female, male, or transgender	0.19 (10)	0.15 (4)	0.18 (14)

+Percentages are a function of sex (1996) column totals.

Note: Cross-Sex identified refers to respondents who endorsed a female natal sex and a male current gender identity, or a male natal sex and a female current gender identity.

Cisgender = Non-Gender Minority (concordant maternal-reported natal sex in 1996 and self-reported current gender identity in 2010).

**Table 3:** **Phase 1: Sociodemographic and other gender-related characteristics among U.S. young adults in the growing up today study 1**

	Gender minority (n = 26)	Cisgender (n = 7805)			Total sample (n = 7831)
	*Mean (SD)*	*Mean (SD)*	*t-test (df)*	*p-value*	*Mean (SD)*
**Age (2005) (n = 7068)**					
Age in Years	20.8 (1.9)	20.7 (1.7)	−0.39 (7066)	0.70	20.7 (1.7)
	*% (n)*	*% (n)*	*χ2 (df)*	*p-value*	*% (n)*
**Race/Ethnicity (1996) (n = 7831)**					
White/Caucasian (Non-Hispanic)	100.0 (26)	96.4 (7527)	0.96 (1)	0.33	96.4 (7553)
People of color (Racial/Ethnic minority)	0.0 (0)	3.6 (278)			3.6 (278)
**Employment status (2010) (n = 7831)**					
Employed full-time	88.5 (23)	93.3 (7285)	0.99 (1)	0.32	93.3 (7308)
Not employed full-time	11.5 (3)	6.7 (520)			6.7 (523)
**Educational attainment (2010) (n = 7825)**					
College degree or higher	69.2 (18)	80.2 (6252)	1.95 (1)	0.16	80.1 (6270)
Less than college	30.8 (8)	19.8 (1547)			19.9 (1555)
**Recalled childhood gender nonconformity to natal sex ≥ age 11 years (2005) (n = 6700)**					
High (% at or above top decile)	47.6 (10)	15.6 (1041)	16.37 (2)	0.0003	15.7 (1051)
Moderate (% at or above median, below top decile)	28.6 (6)	40.3 (2692)			40.3 (2698)
Low (% below median)	23.8 (5)	44.1 (2946)			44.0 (2951)
**Current socially assigned gender nonconformity to current gender identity (2010) (n = 7708)**					
High (% at or above top decile)	64.0 (16)	17.4 (1340)	37.26 (1)	<0.0001	17.6 (1356)
Not high (% below top decile)	36.0 (9)	82.6 (6343)			82.4 (6352)
**Ever identified as sexual minority in lifetime (2010) (n = 7667)**					
Yes	54.2 (13)	15.4 (1178)	27.39 (1)	<0.0001	15.5 (1191)
No	45.8 (11)	84.6 (6465)			84.5 (6476)

SD = Standard Deviation. Ever Identified as Sexual Minority in Lifetime = Ever Identified as Lesbian/Gay, Bisexual, or Mostly Heterosexual (2010).

Note: The total number of cases included in each bivariate comparison differed due to missing data. The number of cases included in each comparison is indicated in parentheses.

Gender Minority=Cross-Sex Identified, Transgender-Identified, and Do Not Identify as Female, Male, or Transgender.

Note: Sex-specific coding was used (as determined by assigned sex at birth) when categorizing high gender nonconformity. Recalled Childhood Gender Nonconformity: % at or above top decile (score ≥ 9 for males and ≥ 12 for females); % below at or above median, below top decile (score 6-8 for males and 8-11 for females); % below median (score < 6 for males and < 8 for females). Current Socially Assigned Gender Nonconformity: % at or above top decile (score ≥ 5 for males and ≥ 6 for females).

### Phase 2: cognitive testing

Table 4 presents the distribution of assigned sex at birth and current gender identity among participants who completed a cognitive testing interview. All substudy participants reported a natal sex concordant with baseline (maternal-reported) sex.

**Table 4:** **Phase 2: cognitive testing substudy among a purposive sample of U.S. young adults in the growing up today study 1 (n = 39)**

	Natal sex (2013)	
	Female (n = 21)	Male (n = 18)
**Current gender identity (2013)**		
Cisgender female (n = 16)	16	0
Cisgender male (n = 14)	0	14
Gender minority (Cross-sex identified, transgender, or do not identify) (n = 9)	5	4
Female	0	2
Male	2	0
Transgender	2	1
Do not identify as female, male, or transgender	1	1

Note: Cognitive testing with 34.6% (9/26) of gender minority respondents identified by the gender identity question in 2010.

+Percentages are a function of the assigned sex at birth column totals.

#### Survey item 1: natal sex

##### Item interpretation

All participants were clear that the natal sex item was asking what was on their original birth certificate and the sex they were designated at birth. All participants also mentioned that “physical anatomy” was used to designate sex on birth certificates, for example: “Penis or vagina. Male or female”. Participants frequently described their process interpreting the item as “automatic” to indicate ease of response: “I didn’t really even think about it. It was just kind of – geez, automatic reaction” (cisgender female). Gender minority participants correctly identified that the item was asking about their assigned sex at birth on their original birth certificate: “I can’t change my birth certificate in the state where I was born, so what’s on my birth certificate now is what’s on it originally” (male birth sex, female-identified).

##### Item clarity

All participants stated that they found the question clear and easy to answer. Participants most commonly used the phrase “straight forward” (77.8%, n = 28); others described it as “obvious” and “an easy question”. Three cisgender male participants (21.4%) felt the phrasing of the question stem was awkward: “It was an awkward question, just the way it was asked. But it’s very clear and concise. Just the grammar is awkward”. However, the question did not lead to inaccurate response: 100% of participants were correctly classified in their natal sex. None of these participants suggested changing the item. No cisgender female or gender minority participants remarked on phrasing awkwardness.

##### Response options

All participants felt the response options “Male” and “Female” were acceptable. Several participants commented that they could not think of any other response options. One participant described being diagnosed with a medically intersex condition but did not think an “intersex” option was needed because male and female reflect the options on a birth certificate.

##### Emotional response

The most common emotional response to the question was laughter, especially for participants who had not given thought to their sex at birth. A cisgender male respondent described his thought process: “I honestly didn’t need to put a whole lot of thought into that one (laughter)”. Two participants —one had skipped the gender identity item in 2010 and the other had not—expressed negative emotional reactions: “Uh, probably kind of like an eye roll type thing in my mind, ’cause uh -- I mean this whole next question got into the whole transgender, all this other stuff. I’m not a big proponent of that, I’ll say. (laughs)” (cisgender male).

#### Survey item 2: current gender identity

##### Item interpretation

Most participants (82.1%; n = 32) interpreted the gender identity item to be about their *internal* sense of gender, which they recognized may or may not be the same as a person’s birth sex. As one cisgender female said: “I assumed that it was a question getting at, you know, there are people that are anatomically female, but identify *mentally, psychologically*, all of those things, with a different gender, or with one of those other options”. Cisgender participants used phrases like “inside feeling”; transgender participants said “how I pictured myself, mentally” and “how I *actually* view myself”. The remaining seven participants were all cisgender and interpreted the item to be about sex: “When I was deciding what to answer, this not really a decision. I just put the answer as male, because I’m -- I don’t know -- male”. (cisgender male).

##### Item clarity

Participants described the question as “straight forward” and “obvious”. One cisgender female summarized what many participants also described: “I checked female. This was not confusing for me because I identify as female. I knew which box was for me”.

##### Response options

Cisgender participants were clear about the response options “Female” and “Male”, although some had difficulty articulating what the term meant to them. One cisgender male stated: “What was I thinking about? I don’t know how to answer that question. I was just thinking, ‘I’m a male. I have always been a male, and I have male parts”. Transgender participants saw “Male” and “Female” response options as a combination of biology and socialization. One participant said: “It’s a combination of sex organs, hormone structure, how you present yourself. All that”. (female birth sex, transgender-identified).

For the most part, cisgender participants understood the response option “Transgender” as a person’s internal sense of gender being different from their physiological sex: for example, “people who were born a male or a female wanting to become the opposite sex”. The key challenge identified by transgender and cross-sex identified participants was selecting the best response option to describe their gender identity. Transgender participants described “switching” responses or trying to decide between two responses, most commonly whether to check “Transgender” or to check “Male” or “Female”. For example: “I originally picked transgender, and then went back and picked female… It did say ‘feel’, so I was pretty sure that it, you know, meant, like, identify with and stuff” (male birth sex, female-identified). Another transgender participant commented: “There were only two options, obviously, that I felt like applied, and it was either male or transgender… I just chose male because like that's how I identify in the world, really. I don't – like transgender, it kind of puts me in this other category that is not equal to, but like less than”. (female birth sex, male-identified). Another transgender participant selected “Transgender” rather than “Male” to make themselves visible as a transgender person in certain settings: “I self-identify as male. But I will say I’m trans for clarity, like here, or like in a medical setting just to be sure people know what’s up…. It made sense to check trans” (female birth sex, transgender-identified).

The majority of cisgender participants (83.3%; n = 25/30) were not sure what was meant by the response option “Do not identify as female, male, or transgender”. Participants had varied ideas of who this category might include, such as: “a nonconformist”; “pan-sexual maybe”; “Um -- hermaphrodite?” An incomplete understanding of the category, however, did not affect accuracy of participant response. It was clear that participants knew which response to check for themselves, stating, for example: “The other options just didn’t apply to me” (cisgender female). Transgender participants interpreted the “Do not identify” response option as “genderqueer”. One participant described it as a response for a younger generation: “I do believe that the youth term is ‘genderqueer’… They don’t want to be determined on whether they’re male or female” (male birth sex, transgender-identified). The general consensus was “You’d know if you qualified for that [box]” (male birth sex, female-identified).

Two participants selected “Do not identify”. Both participants described feeling their gender expression was nonconforming or gender variant and stated they did not identify with gender categories: “I just don’t relate to ‘transgender’ at all. I haven’t come up with a cool new term for me or something like that… it doesn’t relate to me at all” (male birth sex, do not identify).

##### Completeness of response options

Only one cisgender participant commented on the number of response options: “Um, I’m surprised, actually, that there were only four options. (laughs) Yeah…I know people that might have preferred different words or different terminology” (cisgender male). The majority 77.8% (n = 7/9) of gender minority participants liked the four closed-ended response options that were provided, stating, for example, “I feel like it had a response for everybody in a way that was worded appropriately. For me, I felt like it kind of covered everything” (female birth sex, transgender-identified). Two participants suggested additional response options, although both found the current response options to be acceptable. The participant with a medically intersexed condition suggested adding another response option “intersex”. A second respondent suggested modifying “transgender” and breaking it into two separate categories “transgender, female-to-male” and “transgender, male-to-female” in order to be more specific.

One respondent wanted a write-in response (to write-in “genderqueer”): “I would love a write-in. I was a little bit, like, disappointed, but what are you gonna do? It’s like, it comes in small steps, and so, even the fact that they created a state for transgender and created a state for I don’t pick any is, you know, very different from how it was when I was a younger kid” (female birth sex, do not identify). However, the other respondent who selected “Do not identify” felt an open-ended write-in would make it more difficult to answer the question.

##### Emotional response

Overall acceptability was high: “You know, when I saw it, I thought that was awesome” (cisgender male). This was true even for participants less knowledgeable about transgender issues: “It was kind of a shock, but I was not offended. I didn’t mind answering at all” (cisgender female). Participants also described the question as being about diversity: “My thought is that it’s normal, because, uh, there’s a lot of different kind of people out there. (laughter)” (cisgender female).

Two cisgender participants expressed negativity but were not offended by the question: “I feel like there’s a lot of political sensitivity and maybe because I am in the majority, I feel like it’s a little bit overused. I don’t find the question offensive, I just sometimes think maybe we are a little bit too concerned with how people feel rather than reality. But that’s easy to say that when you feel just like the rest of the majority” (cisgender female). Gender minority participants expressed excitement about the gender identity survey item, such as this respondent: “I mean, it’s been -- been years to get to that point, so it’s kind of a big deal. And, you know, I’m excited about where it’s going” (female birth sex, do not identify).

## Discussion

Using a large prospective cohort study of U.S. young adults and a unique mixed-methods design, this study provided information about the validity of two brief items used to measure natal sex/gender status via a two-step method. Analyses of GUTS1 data found good evidence in support of measure construct validity. As hypothesized, compared to cisgender males and females, gender minority participants had higher levels of recalled childhood gender nonconformity and current socially assigned gender nonconformity and were more likely to have identified as not completely heterosexual in their lifetime. There were no major problems identified in cognitive testing interviews in any of the four areas typically involved in question-response processes that might negatively influence the quality and accuracy of data from these survey items [35]: item comprehension; retrieval or recall difficulty; judgment, including biases or selective editing; and response, including having adequate response options. Participants understood the natal sex and gender identity items and correctly interpreted both the question stems and response options. The items performed as anticipated and were acceptable to the vast majority of participants. In addition, the natal sex item in cognitive interviews correctly classified all participants according to maternal-reported sex at baseline.

Overall, 0.33% of the GUTS1 cohort 2010 participants were gender minority. This prevalence is similar to estimates of gender minority population size [37]. The largest gender minority group was comprised of young adults endorsing the response option “do not identify as female, male, or transgender” (14 of 26 participants). This finding, in conjunction with prior research [38, 39], suggests that it is important to offer a non-binary response option beyond female, male, or transgender in gender minority-inclusive data collection efforts, particularly for young adults.

Findings should be considered alongside several limitations. Given that the GUTS cohort is largely homogenous in terms of age (restricted to young adults), and racial/ethnic and socioeconomic status (largely white, all children of nurses), additional research with more diverse samples is needed to further validate the assigned sex at birth and current gender identity items. Moreover, we recommend that the survey items be translated and cognitively tested in additional languages in order to monitor the health of diverse transgender populations in the U.S. and globally. Also needed is study replication in samples that are testing naïve. GUTS1 participants have completed closed-ended health surveys annually or biannually since late childhood or early adolescence; thus, there may be testing effects [40] such as higher levels of comfort with answering novel survey questions. An additional limitation is that the data analytic sample represents only the subgroup of GUTS1 participants who were retained during years of follow-up from 1996 to 2010. Lastly, the low response rate for cognitive testing interviews means that there may have been gaps in the range of perspectives included in the study; however, we interviewed to saturation suggesting that we captured key themes.

An additional consideration is that there are many sources and types of data that permit research in gender minority health in addition to population research. Research projects may require different measures than those tested in the current study, depending on the data source, sampling method, research questions, and aims of the study [13, 41]. For example, a large community-based study restricted to gender minority people would require a wide range of diverse gender identity and expression response options in order to ensure culturally competent and gender affirming data collection [38]. To ensure scientific and conceptual rigor in gender minority research, it is important that researchers carefully consider which dimensions of sex and gender are important to the research question, and how to best operationalize these in designing studies.

Future research on gender minority health would benefit from designing and testing survey questions to accompany natal sex/gender status via skip patterns [13]. For example, validated gender affirmation questions could be asked of gender minority respondents, such as dimensions of social (e.g., pronoun, name, living full-time or part-time in one’s gender), legal (e.g., gender documentation), and medical (e.g., cross-sex hormones, types of surgery) transition. These questions would increase understanding of sex- and gender-linked pathways in gender minority health.

## Conclusions

We recommend that these two brief measures be used to monitor gender-related health disparities through cross-sectional and longitudinal population research in the U.S. [8]. For example, national cohort surveillance systems, such as twin registries and the U.S. Veteran’s Administration medical records system, represent a largely untapped and cost-effective resource to prospectively study transgender population health. This validation study provides new tools for incorporating natal sex/gender status into health research.
